# State-of-the-Science Review of Non-Chemical Stressors Found in a Child’s Social Environment

**DOI:** 10.3390/ijerph16224417

**Published:** 2019-11-11

**Authors:** Kathleen Hibbert, Nicolle S. Tulve

**Affiliations:** U.S. Environmental Protection Agency, Office of Research and Development, National Exposure Research Laboratory, 109 TW Alexander Drive, Research Triangle Park, Piedmont, NC 27709, USA; tulve.nicolle@epa.gov

**Keywords:** non-chemical stressors, public health, exposure, children, social determinants of health

## Abstract

*Background*: Children are exposed to chemical and non-chemical stressors from their built, natural, and social environments. Research is needed to advance our scientific understanding of non-chemical stressors, evaluate how they alter the biological response to a chemical stressor, and determine how they impact children’s health and well-being. To do this, we conducted a state-of-the-science review of non-chemical stressors found in a child’s social environment. *Methods*: Studies eligible for inclusion in this review were identified through a search of the peer-reviewed literature using PubMed and PsycINFO. Combinations of words associated with non-chemical stressors and children were used to form search strings. Filters were used to limit the search to studies published in peer-reviewed journals from 2000–2016 and written in English. Publications found using the search strings and filters went through two rounds of screening. *Results*: A total of 146 studies met the inclusion criteria. From these studies, 245 non-chemical stressors were evaluated. The non-chemical stressors were then organized into 13 general topic areas: acculturation, adverse childhood experiences, economic, education, family dynamics, food, greenspace, neighborhood, social, stress, urbanicity, violence, and other. Additional information on health outcomes, studies evaluating both chemical and non-chemical stressors, and animal studies are provided. This review provides evidence that non-chemical stressors found in a child’s social environment do influence their health and well-being in both beneficial (e.g., salutatory effects of greenspace and social support) and adverse (e.g., poor relationships between health and selected non-chemical stressors such as economics, educational attainment, exposure to violence, stress) ways. *Conclusions*: This literature review identified a paucity of studies addressing the combined effects of chemical and non-chemical stressors and children’s health and well-being. This literature review was further complicated by inconsistencies in terminology, methodologies, and the value of non-chemical stressor research in different scientific disciplines. Despite these limitations, this review showed the importance of considering non-chemical stressors from a child’s social environment when addressing children’s environmental health considerations.

## 1. Introduction

Children are exposed to both chemical and non-chemical stressors from their built, natural, and social environments [1]. Non-chemical stressors are factors found in these environments, including physical factors (e.g., noise, temperature, and humidity) and psychosocial factors (e.g., poor diet and illicit drug use) [1]. The social environment can include elements such as exposure to violence, greenspace access and use, social support, access to education, psychological stress, family income, socioeconomic status, neighborhood quality, acculturation, and food sources. Current research suggests that non-chemical stressors modify the biological response to chemical stressors, thereby impacting children’s health and well-being. Children may be more vulnerable to the combined effects of chemical and non-chemical stressors due to their physiology (e.g., metabolic rate, surface-area-to-body-weight) and lifestage-specific activities and behaviors (such as mouthing, crawling or playing close to the ground, playing outdoors) when compared to adults. The combination of their continual physiological development, the nature of their motor, cognitive, and life course developments, and reliance on a caretaker (lack of independence) are all reasons for a child’s increased vulnerability to the combined effects of chemical and non-chemical stressors [1,2,3,4,5].

As the exposure assessment paradigm has shifted from single chemicals to multiple chemicals to mixtures, the scientific community has realized the need to include non-chemical stressors in studies evaluating chemical stressors [6,7,8,9,10,11,12]. In 2011, a workshop at the Society for Toxicology annual meeting resulted in work addressing the inclusion of non-chemical stressors into cumulative risk assessment [13] followed by additional workshops on cumulative risk and multiple exposures [11]. Previous work on non-chemical stressors and public health has typically excluded considerations of chemical exposures [14,15,16,17,18,19].

Research on non-chemical stressors is needed to advance our scientific understanding of non-chemical stressors, and how they alter the biological response to a chemical stressor, in regard to their impact on children’s health and well-being. Studies have evaluated how non-chemical stressors directly affect animal health and well-being; for instance, a study that looked at the health effects of cows from crowding and sleep deprivation [20]. Other studies were designed to look at indirect effects on health and well-being, such as a study that determined if exposure to greenspace influenced physical activity [21], thus, influencing health. There are very few studies that address outcomes from the combination of non-chemical and chemical stressors, or the interaction effect(s) of non-chemical and chemical stressors.

The primary objective of this research is to conduct a state-of-the-science review of non-chemical stressors found in a child’s social environment that might impact individual health. This review synthesizes many studies, included through predetermined criteria, into general topic areas. The secondary objective of this review is to identify which topic areas have begun to research the relationships between chemical and non-chemical stressors, and how the relationships may impact health outcomes. This review will help to identify possible gaps in the science of non-chemical stressors and inform future research design.

## 2. Materials and Methods

### 2.1. Literature Search

Studies eligible for inclusion in this review were identified through a search of the peer-reviewed literature using PubMed and PsycINFO. Combinations of words associated with non-chemical stressors (e.g., non chemical AND (stressor OR factor)) AND children were used to form search strings. Initial search strings included the general terms non-chemical stressor OR non-chemical factor AND health. Following the initial search, additional terms (i.e., psychosocial or social determinants or social environment) AND child* AND health were added to the search strings. Finally, search strings included specific non-chemical stressors (e.g., socioeconomic or exposure to violence or social support or acculturation or food access or overcrowd or urbanization or greenspace) AND child* AND health. Filters were used to limit the search to studies published in peer-reviewed journals from 2000–2016 and written in English.

### 2.2. Study Selection

**Figure 1:**
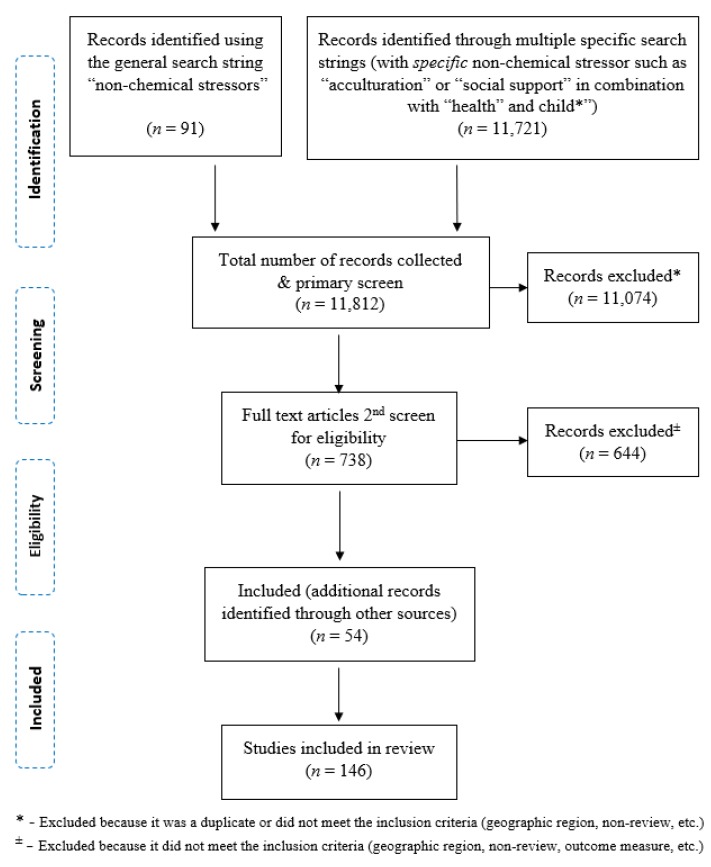
Diagram of record selection, eligibility, and inclusion; adapted from Preferred Reporting Items for Systematic Reviews and Meta-Analysis (PRISMA) [22].

The Preferred Reporting Items for Systematic Reviews and Meta-Analysis (PRISMA) [22] method was used for the literature search phase (Figure 1). Publications found using the search strings and filters went through two rounds of screening. During the first round, titles and abstracts were screened and selected based on their relevance and whether they met the inclusion criteria listed below. In the second round, full text articles of selected abstracts were retrieved and reviewed. For articles that met the inclusion criteria, the references were reviewed for additional relevant citations.

#### 2.2.1. Inclusion Criteria

To be included in this review, studies needed to meet the following criteria.

(1) experimental or observational studies;

(2) study was designed to evaluate a non-chemical stressor found in the social environment of the participants;

(3) study evaluated a non-chemical stressor as a variable that impacts health;

(4) study cohort was located in the United States, Canada, or Europe;

(5) non-chemical stressor classification used the Tulve et al. [1] conceptual framework, which does not include intrinsic biological factors, biological pathogens, or activities/behaviors.

#### 2.2.2. Data Extraction and Synthesis

Information extracted from the eligible studies included: article identifiers (author(s), year), study characteristics, subject demographics, non-chemical stressors, chemical stressors, outcome measures, and health impact results. After this information was extracted, non-chemical stressor variables from articles were identified and categorized into general topic areas for further analysis. Mutual variables from articles were then quantified, and descriptive analyses were completed on the extracted information. Table 1 summarizes the characteristics of the studies included in this review.

## 3. Results

### 3.1. Results of General Topics

**Figure 2:**
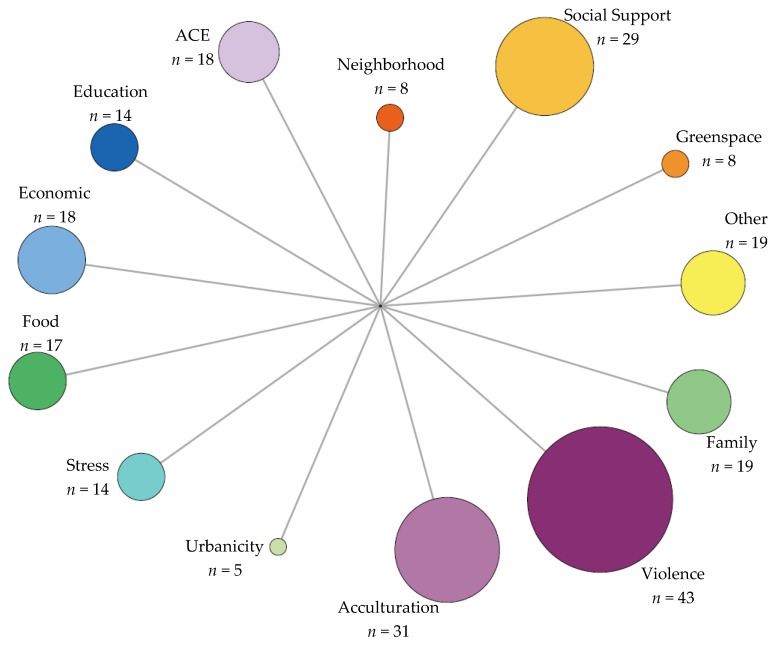
Non-chemical stressor general topic categories by frequency. -Size of circle represents frequency of times topic is investigated.

A total of 146 studies met the inclusion criteria. From these studies, a total of 245 non-chemical stressors were evaluated. The non-chemical stressors were organized into 13 general topic areas: acculturation, adverse childhood experiences (ACE), economic, education, family dynamics, food, greenspace, neighborhood, social, stress, urbanicity, violence, and other (other included several variables that were seen once or twice that were considered non-chemical stressors but did not fit into another general topic area) (Supplemental Materials; Table S1). Figure 2 displays the general topic categories resulting from the synthesis of the studies included in this review.

#### 3.1.1. Acculturation

The results describing the relationships between acculturation (*n* = 31) and health are diverse. Roughly 94% of the studies reported that acculturation impacted health. Of those, almost 38% found that higher acculturation was related to beneficial health impacts [14,23,24,25,26,27,28,29,30,31,32], whereas 48% found that higher acculturation was linked to adverse health impacts [17,33,34,35,36,37,38,39,40,41,42,43,44,45]. Results also indicated that 14% of the studies had mixed results [46,47,48,49] and 6% had either non-significant results [50] or suggestive results from qualitative analyses of focus groups [51]. Interestingly, culture of origin did not offer an explanation for the variation on whether the statistical relationship was positive or negative. Of the 31 studies that considered the health impact from acculturation measures, none jointly investigated a chemical exposure.

#### 3.1.2. Adverse Childhood Experiences

Eighteen articles studied adverse childhood experiences and all reported negative impacts on health and well-being [52,53,54,55,56,57,58,59,60,61,62,63,64,65,66,67,68,69]. These studies included violence, as well as other types of adverse childhood experiences (e.g., divorce, parental mental illness). In 1995, Kaiser Permanente and the Centers for Disease Control and Prevention (CDC) jointly conducted one of the largest investigations of childhood abuse and neglect and later life health and well-being, known as the CDC-Kaiser ACE Study. The CDC-Kaiser ACE study included a retrospective survey and longitudinal tracking of study participants that evaluated health and well-being outcomes against multiple childhood experience variables, such as physical abuse, neglect, substance abuse, or living with a person with mental illness in the home. The CDC-Kaiser ACE study provided strong evidence that a significant positive relationship existed between childhood exposure to multiple adverse childhood experiences (e.g., non-chemical stressors) and adult health and well-being [52,56,57].

Certain studies identified a relationship between exposure to violence or adverse experiences and health impacts that mimicked a dose-response relationship. Those studies offered evidence that the more adverse experiences a participant had, or the more types of violence experienced (physical, sexual, or physical and sexual) or more time exposed to adverse experiences or violence, increased the negative health impact resulting in risky behavior, general adverse health, telomere erosion, comorbid depression, chronic pain, hypertension, neurological responses, and heart disease [52,56,57,68,70,71,72,73]. Of the eighteen studies on adverse experiences by children included in this review, only one (<6%) investigated the relationship between a chemical stressor and a non-chemical stressor and their combined impact on a child’s health. Stein et al. [69] identified not only the negative associations between total adversities and child cognition, but also that associations were stronger when higher levels of organophosphate metabolites were higher, with gender variations.

#### 3.1.3. Economic

Previous research offers evidence that socioeconomic disadvantages (e.g., lower income, less educational attainment) are linked to poorer general health, higher morbidity and mortality rates [23,74,75,76,77], and more susceptibility to chemical exposures [78]. Additionally, research has shown that socioeconomic and sociodemographic characteristics affect choices that result in increased chemical exposures [79].

For this review, economic measures included wealth, income, disposable income, or an index such as socioeconomic status (SES) or position (SEP), or poverty. Economic influences on health showed consistent associations across all studies (*n* = 20) and several were significant (*n* = 17). The lower the income, wealth, or resources, the higher the likelihood of negative health impacts or prevalence of illness studied [18,23,26,31,39,80,81,82,83,84,85,86,87,88,89,90,91]. These measures were also studied at different scales. Of the studies, 70% reported measures at the individual/household level [18,23,26,39,80,82,83,84,85,87,88,89,90,91], while 25% studied variables at the community/neighborhood level [31,86,92,93,94]. Only one study reported economic measures at a country level [81]. Several studies evaluated whether effects from income were seen as gradients (e.g., a correlational relationship between the level of income and the severity of the health impact) [23,26,82,83,84,92]. Descriptive analyses suggested that 85% of the articles reported a negative relationship between an economic influence and a health outcome. The remaining studies had mixed results depending on the geographic scale [93] and the other variables included in the model [94]. Unlike most of the social stressors in this review, three studies (15%) involved chemical stressors (cigarette smoke, chlorpyrifos, and industrial pollution) in their research design [86,89].

#### 3.1.4. Education

Evidence in the literature shows an association between caregiver educational attainment and child health [95]. Additional evidence shows a positive correlation between educational attainment, mortality, and life expectancy [96,97]. There is also research that offers evidence that education level can be a predictor of chemical usage in a household environment [79], as well as a component of SES [87,88].

Fourteen articles evaluated the relationship between educational attainment and health and well-being, with seven articles linking higher educational attainment to positive health impacts [27,29,88,96,97,98,99] and six articles linking lower educational attainment to negative health impacts [33,81,87,94,100,101]. Ultimately, both reporting scenarios conveyed a positive relationship between education level and quality of health. Although 13 articles offered evidence that lower educational attainment for the participant or caregivers had negative impacts on health and well-being, one article found that depending on race and disease, some instances were found where higher parental educational attainment increased the likelihood of disease for the child [102]. None of the educational attainment research included in this review looked at effects on health and well-being from a combination of chemical exposures and educational attainment.

#### 3.1.5. Family Dynamics

Nineteen articles evaluated family dynamics, such as, family communication and relationships [24,103,104,105], familial support and involvement [80,84,106,107,108,109], and family structure or environment, such as, stability [87], siblings [110], parental structure (single vs. couple) [16], or parenting style [24]. Some of the research looked at familial physical [47,94] and well-being characteristics [71,111,112] that might influence a child’s social environment. Stronger or more positive familial support and better communication and relationships resulted in a positive impact on health and well-being [80,103,104,105,106], while unhealthy relationships and lack of family support and time together showed significant negative impacts on health and well-being [84,87,107,108]. Family structure (such as fewer siblings [110] or a single parent-household [16]) also showed a negative impact on child health and well-being. Living in a social environment with a depressed mother (or parents) [71,111,112], parents using a democratic parenting style [24], or overweight parents [47,94] had a negative impact on a child’s health and well-being. None of the research included in the family dynamic category studied effects on health and well-being from a combination of chemical exposures and non-chemical stressors.

#### 3.1.6. Food

Previous research has been conducted involving relationships between food and health, including topics on food insecurity [113,114,115], food acculturation [14,34,116], and income [117,118]. Some research focuses on food choices, such as fast food habits [119], organic vs. non-organic, or farmers market habits [120,121], and food choices which emulate the habits of one’s caretaker [122]; while other research has focused on such things as community exposure to agriculture [123,124,125] or acculturation of caretakers [27] to explain variations in fruit and vegetable intake for children. Food sources (e.g., fast food, convenience store, supermarket, farmers marker), access to food, and food preparation are derived from the social environment of a child and all contribute to children’s health. Research has identified trends in food location, food source, and purchasing habits leading to sources of energy, sugar, sodium, and nutrients [126,127,128,129,130,131,132]. There is also a large body of research that identified food as the source for exposures to toxicants (i.e., flame retardants, pesticides, insecticides) [121,133,134,135,136,137].

Several studies included in this review (*n* = 17) evaluated food in a social context. Some of the research studied food (in)security [31,35,113,120,138,139,140,141], food access [93], and skipping/eating patterns [32,37]. Additional research studied health impacts resulting from food choices. For example, Lumia et al. [142] showed the advantageous health impacts from early life exposure to fish. Chen et al. [25] and Liu et al. [38] reported on the undesirable health impacts from a poor diet. Drewnowski et al. [143] studied supermarket access. One study compared lunches purchased at school versus lunches brought from home and found that both included negative choices that could adversely impact health [132]. Most of these studies offered evidence that poor diet, lack of food, and meal skipping resulted in both short- and long-term negative health effects, such as obesity, hyperactivity, depression, hypertension, and improper nutrient intake [25,31,37,38,132,138,139,141,143]. Only one of the seventeen studies (<6%) considered both a chemical and non-chemical stressor by looking at air pollution from oil refineries and industrial settings, and access to food [93].

#### 3.1.7. Greenspace

For this review, greenspace, waterways/rivers/lakes/oceans (anything considered bluespace), and natural or restorative environments were classified as greenspace. Greenspace is identified as “urban nature” or “residential greenspace”, and includes parks, fields, forests, gardens, and yards [94,103,110,144,145,146,147]. This review identified studies (*n* = 8) that researched greenspace locations and uses through a social lens. From those studies, various greenspace-related non-chemical stressors were identified (residential greenspace, urban greenspace, greenspace use, proximity to park, perceived lack of greenspace, coastal proximity, neighborhood greenspace, time spent in greenspace) as well as additional non-chemical stressors that were considered within the research (family relations, neighborhood quality, social capital, rural/urban, SES, number of siblings) [94,103,110,144,145,146,147]. Five of the greenspace non-chemical stressors investigated (62%) offered evidence of salutary health effects, which included increased social capital, improved asthma outcomes, higher esteem, and better emotional well-being [103,110,144,145,147]. Results associated with the remaining three non-chemical stressors were either inconclusive [146] or not significant [94,110]. Greenspace can be described as a component of both the natural and social environments. For example, the Montreal Study followed immigrant children and their families, and found that social support was significantly correlated with “urban nature” and negatively correlated with emotional stress [144]. Additional greenspace research has shown that higher greenspace use results in positive impacts on health-related quality of life and friendships [110]. Greenspace use has also been correlated with reducing aggressive behavior and increasing emotional well-being among children between the ages of 7 and 18 years old [144,147].

Chen et al. [103] reported a significant interaction effect between the quality of the parent–child relationship and residential greenspace. They showed that as the relationship improved, residential greenspace was more strongly associated with better asthma control [103]. Other studies found that urban greenspace usage was related to quality of life for children [110] and lowered aggressive behaviors in adolescents [147], while perceived lack of greenspace was associated with increased body mass index (BMI) [94]. Hordyk et al. [144] used a hermeneutic phenomenological approach to observe that urban nature strengthened social cohesion for immigrants and minimized emotional stress. McCracken et al. [110] found that the use of greenspace influenced participants’ self-esteem. Another study found that being close to a *preferred* park was influential for both usage and health, while being close to *a* park was not [145]. Studies did not consider a combination of non-chemical and chemical exposures when analyzing greenspace and health effects.

#### 3.1.8. Neighborhood

Eight studies evaluated whether neighborhood characteristics influenced health outcomes. Some evidence exists in the literature to suggest that neighborhood disorder [50,148], neighborhood disadvantage [149], low quality of neighborhood [150], and neighborhood problems [108] all adversely impact health, such as via substance abuse, asthma, pulmonary function, or general well-being, to name a few. However, two studies that looked at neighborhood disadvantage [80,93] did not report adverse health effects. Another study on neighborhood quality (criminal activity, substance usage, and vandalism) [147] did not report any adverse health impacts. Two studies included both chemical and non-chemical stressors in their analysis [93,148]. These studies both included considerations and analysis for the exposures from air pollution (NO_2_ and air toxic emissions); however, neither study identified interaction effects between the chemical and non-chemical stressors on health and well-being.

#### 3.1.9. Social Support

Social support is described as a social network of peers and/or family that a person may rely on for emotional support throughout the lifecourse [108]. Social support has been shown to improve physical functioning of the ill and reduce depressive symptoms [151,152], as well as improve treatment compliance [153]. Social support is generally analyzed by measuring attributes, such as visiting neighbors, neighborhood involvement, or number of friends [18,92,110,154,155]. Social support is also seen as a potential by-product of living near “like” people, such as immigrant enclaves or participation in outdoor activities [110,144].

Social support (*n* = 29) as a non-chemical stressor often presented as a salutary measure/variable if the social support existed and was positive. For example, even in an economically burdened neighborhood, such as Baltimore, MD, USA, a belief of having a caring adult in the home was correlated with a child’s hope [152]. Other research that reported findings of salutary impacts on health and well-being confirmed that both perceptions of social support and actual social support had beneficial health impacts [18,45,63,67,82,104,110,151,152,154,155,156,157,158,159,160,161,162,163]. Similarly, research offered evidence that low quality social relations, lacking social support, or high social disadvantage have adverse health impacts including, but not limited to, general mental/physical health problems, attention deficit hyperactivity disorder (ADHD), obesity, and cardiometabolic disease [17,64,107,164,165,166]. Five studies (17%) had either inconclusive or non-significant results for social support impacts on health and well-being [90,92,108,162,167]. Caldwell et al. [157] was the only study that looked at a non-chemical stressor (social support) and a chemical exposure (prenatal exposure to ethanol). The impairment seen in the animal test groups exposed to alcohol was ameliorated with communal living (vs. isolation) [157].

#### 3.1.10. Stress

**Figure 3:**
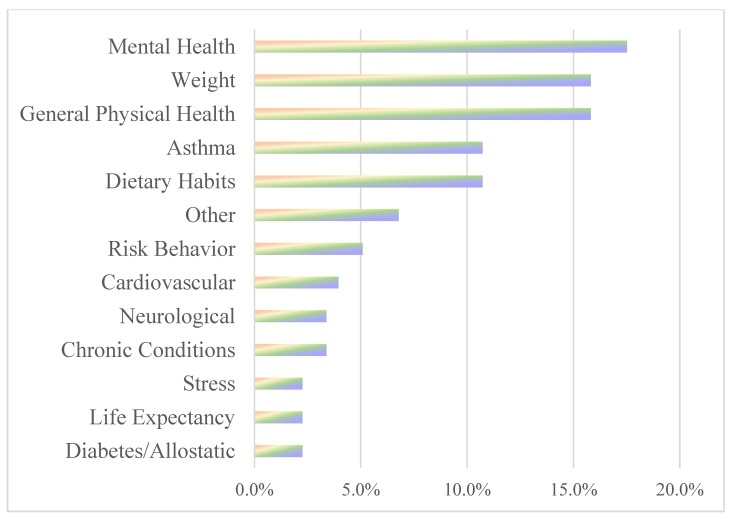
Total percent of articles reporting on health outcomes, measurements, or behaviors.

Studies on stress (*n* = 14) as a non-chemical stressor consistently (86%) had findings of a negative relationship between stress and health and well-being, such that high levels of stress were inversely associated with health outcomes [7,48,60,67,68,168,169,170,171,172,173]. Only two studies indicated either not significant or mixed results [4,174]. Five of the studies (28%) included a chemical exposure in addition to the non-chemical stressor in the analysis [4,7,170,171,175]. Clougherty et al. [170], Clougherty et al. [171], and Cowell et al. [172] found that adverse health outcomes were heightened or exacerbated with the presence of the non-chemical stressor. In an animal study that analyzed footshocks (stress) and chlorfenvinphos, the results found that, independently, the insecticide or the footshocks (stress) had adverse health outcomes, but stress appeared to have a protective effect that diminished the adverse health outcome from the insecticide when the exposure to stress (footshocks) preceded the insecticide exposure [175]. In addition to stress as a non-chemical stressor, stress was identified in a few of the studies as a health outcome or measure which included measures of general stress, cortisol levels, cortisol reactivity, and persistence of cortisol [66,106,144,175]. Stress as a health outcome is briefly acknowledged in the health outcomes section and Figure 3; however, this section is addressing stress as a non-chemical factor.

#### 3.1.11. Urbanicity

This review identified five articles that assessed geographic environments (urbanicity: e.g., rural, suburban, urban) on health and well-being. Rural urbanization encompasses stressors, such as a shift from agrarian to industrial society factors, caregiver education, sanitation practices, food options (availability and behaviors), and health care access and practices. Many stressors associated with urban development may have both individual and community level effects. Previous studies have shown that children in urban areas grow faster than those in rural areas [176], offering evidence that an urban setting can influence physiological changes occurring throughout a child’s lifecourse. Other studies show significant differences in food quality consumption [177], asthma prevalence [178], and general health conditions [179] between urban and rural regions.

The studies included in this review highlighted research that showed that both higher urbanicity [81,180] and lower urbanicity [27,100,146] were associated with negative health impacts depending on the health outcome measured. For example, Protano et al. [100] and Wood et al. [146] saw associations between lower urbanicity and increased body weight/obesity. Erinosho et al. [27] identified a link between rural environments and consuming less vegetables. Breslau et al. [180] and Chai et al. [81] both identified stronger correlations between high urbanicity and exposure to violence, which led to indirect impacts, such as, but not limited to, higher incidences of post-traumatic stress disorder (PTSD) in females. In addition, recent research exists on non-chemical stressors (acculturation, meal patterns, food insecurity, exposure to violence, and social determinants) in predominantly urban or rural populations, but does not test for urbanicity as a variable of influence [37,113,181,182], emphasizing the need for more studies that consider urbanicity as a non-chemical stressor in future studies. None of the studies included in this review researched urbanicity and chemical exposures as combined exposures impacting health and well-being.

#### 3.1.12. Violence

When children are exposed to violence, it can lead to health problems throughout the lifecourse. Exposure to violence (ETV) can be direct (being the victim of the violence) or indirect (e.g., witnessing the violence). Types of violence to which a child may be exposed include physical, sexual, verbal/threat, crime, or bullying. ETV can happen on an individual scale with someone familiar or a stranger, a social scale (such as schoolyard bullying or athletic team loyalties), a neighborhood or community scale, or a national scale, which could include political strife, terrorism, and war. Several studies outside of the inclusion criteria for this review indicated that drug and alcohol misuse, mental health status, and behavior of adolescents and adults are influenced by early life exposures to violence [183,184,185,186,187,188].

Exposure to violence during childhood resulted in an inverse relationship to a person’s health and well-being. Of the 43 studies included in this review, 93% reported an adverse impact on health and well-being irrespective of the type or scale of the violence [52,53,56,57,58,59,60,62,66,68,70,71,72,73,81,85,101,105,106,145,148,149,161,162,163,168,170,174,180,181,189,190,191,192,193,194,195,196,197,198,199,200,201,202,203,204], whereas 7% of the studies reported either no, mixed, or inconclusive impacts on health and well-being [59,101,191]. Of the studies that evaluated violence as a variable affecting health and well-being, only 7% included a chemical exposure [148,170,181].

#### 3.1.13. Other

Nineteen of the studies did not fit well into the categories and were grouped as “other”. Although race and ethnicity can each be considered non-chemical stressors found in a child’s social environment, this review did not seek to find research on race or ethnicity because this research used the construct published by Tulve et al. [1] as a foundation for study and search design. There were, however, four studies in this review that identified race, ethnicity, or ancestry as an interactive variable which had significant impacts on the health outcomes for children [33,45,102,192]. There were three studies that identified geographic-type influence (e.g., area of city [180], crowding [148], country of origin [32]) which had significant negative impacts on children’s health. On the contrary, Waters et al. [112] did not find overcrowding to have a negative effect on health and when Drewnowski et al. [143] measured the effect that distance to (food) market had, they found it to be not significant.

Studies that scrutinized behaviors, such as poor sleep [160] or lower physical activity [38,50], showed significant negative health impacts. Likewise, studies that examined living in household chaos [98], in high turbulence [196], with high child misfortune [64], or with high discrimination [26], all showed significant negative health outcomes. Beaver et al. [80] found that duration of breastfeeding had a significant impact on children’s health.

### 3.2. Additional Results

#### 3.2.1. Health Outcomes

This review did not set inclusion criteria based on health variables measured, so we are able to see what the scientific community is studying regarding health outcomes, indicators, or behaviors, and their linkages to non-chemical stressors. Health outcomes, indicators, and behaviors extracted from the studies in this review were organized into 13 categories: asthma, cardiovascular health, chronic conditions (general), diabetes/allostatic, general physical health, life expectancy, mental health, neurological, risk behavior, stress (In addition, note that in the literature stress is studied as both an independent variable that may impact a health outcome, and as a health outcome or measure which results from non-chemical and/or chemical exposures. Health outcomes for stress might include stress, cortisol levels, or the persistence of cortisol.), weight, and other (e.g., cancer, telomere health, dental, sleep health, memory). Mental health, weight, and general physical health were the most studied, followed by asthma and dietary habits (Figure 3).

#### 3.2.2. Studies Evaluating Both Chemical and Non-Chemical Stressors

Fourteen studies looked at both chemical and non-chemical stressors found in a child’s social environment [4,7,69,86,89,93,148,157,169,170,171,172,175,181] (Table 2). Seven of these studies evaluated responses to stress and either air pollution (NO_2_, black carbon, or concentrated ambient fine particles [169,170,171,172]), pesticides [175], nicotine [4], or alkylphenols [7]. Three were animal studies [4,7,171]. Two of the studies explored linkages between air pollution and community level social stressors [148,181]. The remaining five studies investigated effects from alcohol and early-life rearing conditions (isolated or communal nest of dams) [157], pesticide exposures and neighborhood poverty [86], pesticide exposures and ACEs [69], tobacco smoke and material hardship [89], and oil refineries and neighborhood poverty [93]. Two animal studies suggested salutary effects from the relationship between a non-chemical and chemical stressor [157,175]. Gralewicz et al. [175] reported that exposure to stress induced a cortisol response that had a protective effect against exposure to an organophosphate pesticide (chlorfenvinphos). Another study showed that social support (communal nests) had an ameliorating influence on the negative effect of neurochemical changes as a result of alcohol exposure [157]. Five of the studies that analyzed the effect of non-chemical stressors on a biological response to a chemical exposure stated that the non-chemical stressor either had an exacerbating [89,171], heightened [4,169], or synergistic [170] influence on the health outcome. McCormick et al. [4] also offered evidence that adolescents were especially vulnerable to specific stressors because of their developmental stage.

When studying the links between prenatal exposure to black carbon and community violence, Chiu et al. [181] found an interaction effect on childhood asthma. Shmool et al. [148] found that community level social stressors, such as crime and physical disorder, negatively impacted children’s health independently, but overcrowding and lack of resources modified the NO_2_ levels in the neighborhood.

#### 3.2.3. Animal Studies

Six studies in this review were animal studies (Table 3). Four of the studies analyzed a response to higher stress brought on by footshocks [175], social defeat and overcrowding [173], strangers in their den [4], or predators within observation range [7]. In addition, studies looked at responses to social situations, such as higher socialized living (communal nests) [157] or social isolation [4]. McCormick et al. [4] found negative gender-specific health outcomes during adolescent lifestage exposures to stress, isolation, and nicotine. Clougherty et al. [171], Gergs et al. [7], and Reber et al. [173] had similar findings of significant negative health effects resulting from higher exposures to stress, while Gralewicz et al. [175] found that stress had created a protective effect on the response of hyposensitivity to pesticide exposure. Caldwell et al. [157] found that increased social environments offered significant positive health effects. Overall, it is understood that animal studies can be used to evaluate cause and effect when human studies are not available.

## 4. Discussion

### 4.1. Summary of Findings

Non-chemical stressors have been studied in both the physical and social sciences as both direct and confounding variables. With little exception, the interrelationships between chemical and non-chemical stressors from a child’s social environment are not studied in regard to health and well-being. Reducing this information gap would make relevant scientific contributions to understanding cumulative exposures and risks in a child’s social environment.

Through organizing and synthesizing the current relevant literature this review provides evidence that non-chemical stressors found in a child’s social environment can influence their health and well-being. This review confirmed that adverse relationships exist between health and selected non-chemical stressors including, but not limited to, economic disadvantage, lower educational attainment, exposure to violence, adverse childhood experiences, stress, and urbanicity [24,27,35,56,73,81,83,86,113,143,148,168,170]. On the other hand, this review also identified the salutary effects of some non-chemical stressors, such as exposure to or experience from greenspace and social support [18,91,103,144,152] on health and well-being.

Also identified through the synthesis of extant literature was the paucity of studies evaluating links between chemical and non-chemical stressors and their combined and/or interactive impacts on children’s health and well-being. The existing literature on studies testing non-chemical stressors varies greatly in study design, non-chemical stressor considered, and chemical stressor considered, if one was incorporated. When considering the conceptual framework published by Tulve et al. [1], understanding the interrelationships between chemical and non-chemical stressors is paramount, since people are exposed to both chemical and non-chemical stressors at each lifestage throughout their lifecourse, impacting their health and well-being in countless ways [4,7,86,89,93,148,157,169,170,171,172,175,181].

### 4.2. Limitations

Limitations with this review include the identification of numerous inconsistencies in terminology (e.g., non-chemical stressors, psychosocial stressors, social determinants of health, environmental stressors), methodologies, and the value of non-chemical stressor research in different scientific disciplines. In order to overcome this, the search terms were increased to attempt to capture more studies for the review. Additionally, often the non-chemical stressor being studied is an index for other interchangeable variables (e.g., SES) resulting in more inconsistencies, or can be measured in many non-standardized ways (e.g., stress). This heterogeneity can be problematic for research in public health. An additional limitation includes the lack of interdisciplinary research that bridges the gap between the physical and social sciences, which could strengthen study designs and methodologies. Another limitation would be the possibility there may be additional studies not captured in this review.

## 5. Conclusions

This review presents evidence of the importance of considering non-chemical stressors found in a child’s social environment when addressing children’s health, exposure assessment, or risk assessment. This review also highlights a noticeable dearth in research addressing both non-chemical and chemical stressors, supported by the few studies that showed significant health effects from combined exposures to both chemical and non-chemical stressors. In addition, there is a need for future studies to address the interaction effects of chemical and non-chemical stressors. This review uses existing literature to offer evidence that: (1) non-chemical stressors in a child’s social environment can impact health; (2) the health impact can appear after various latency periods after exposure; (3) the non-chemical stressor can influence the response to a chemical exposure; and (4) very few studies look at the resulting health effects from exposure to both chemical and non-chemical stressors combined. Therefore, this review highlights the importance of including non-chemical stressors found in a child’s social environment when understanding health impacts at each lifestage throughout the lifecourse. It is also important to recognize that not all non-chemical stressors are mutually exclusive. It is important when designing research to not overestimate the burden of the non-chemical exposure by capturing the effect more than once. In this review there were instances of ACE studies that included exposure to violence, there were neighborhood studies that included income measures, and acculturation studies that included social measures. Future research on cumulative exposures including non-chemical and chemical factors needs to acknowledge this to eliminate overestimation of non-chemical stressor effects.

## Figures and Tables

**Table 1:** Summary of the characteristics of the studies included in this review.

**Location of Study**		*n*
	Canada	16
	Europe	19
	United States	102
	Global	3
**Sample Size**		
	<100	21
	100–999	54
	1000–9999	37
	10,000–99,999	22
	>100,000	6
**Study Population**		
	Human	140
	Animal	6

**Table 2:** Summary of the studies included in review that researched both chemical and non-chemical stressors.

Title	BOTH: Chemical Stressor and Non-Chemical Stressor							
	Author, Year	Geographic Location	Chemical Stressor	Specific Chemical(s)	Cohort Age Range	Sample Size	Relationship: Chemical and Non-Chemical Stressors	Impact on Health
[157]	Caldwell, 2015	New Mexico, USA	Prenatal Alcohol Exposure (PAE)	Ethanol	day 40–55	--	mitigating “ameliorated”	High Social = Positive
[169]	Chen, 2008	Vancouver, BC	Traffic pollution	NO_2_	9–18 yr.	73	heightened and increased symptoms	Higher Stress = Negative
[181]	Chiu, 2014	Boston, MA, USA	Urban Pollution	Black Carbon, PM_2.5_	2 yr.	708	only suggested	Community Violence. = Negative
[170]	Clougherty, 2007	Boston, MA, USA	Air pollution	NO_2_	Birth–12 yr. Mean 6.8 yr.	413	synergistic (p1145)	Above Median ETV = Negative
[171]	Clougherty, 2010	--	Air pollution	PM_2.5_ Concentrated Ambient fine particles (CAPs)	--	24	Respiratory response to CAPs exacerbated by chronic stress	Higher Stress = Negative
[172]	Cowell, 2015	Boston, MA, USA	Traffic pollution	Black Carbon	6 yr.	258	Interactive for boys	Higher Stress = Negative
[7]	Gergs, 2013	(Animal)	Alkylphenol	p353-nonylphenol	--	--	Mixed	Higher Stress = Negative
[175]	Gralewicz, 2005	(Rats) Poland	Organophosphate pesticide	Chlorfenvinphos	--	--	Protective	Higher Stress = Positive
[86]	Lovasi, 2011	New York, NY, USA	Pesticides	Chlorpyrifos	36 mo.	266	Independent	Neighborhood Poverty = Negative
[4]	McCormick, 2006	Canada	Nicotine	Nicotine	30–45 d.	132	Mixed	High Stress (social) = Mixed
[93]	Prochaska, 2014	Port Arthur, TX, USA	Exposures from Oil Refineries and Ports	Exposures from Oil Refineries and Ports	--	≈12,028	Not researched	EJ Community = Inconclusive
[89]	Rauh, 2004	USA	Smoking	cigarette smoke	0–2 yr.	226	Exacerbated	Material Hardship = Negative
[148]	Shmool, 2014	New York, USA	Air pollution	NO_2_, SO_2_, PM_2.5_	0–14 yr.	N/A	Not Researched	Higher Violent Crime and Physical Disorder = Negative
[69]	Stein, 2016	Salinas, CA, USA	Pesticides	Organophosphates	0–7yr.	329	Modified negative relationships	Higher adversities = Negative

**Table 3:** Summary of the studies included in review that were animal studies.

Title	Animal Studies							
	Author, Year	Animal	Chemical Stressor	Specific Chemical(s)	Cohort Age Range	Sample Size	Relationship: Chemical and Non-Chemical Stressors	Impact on Health
[157]	Caldwell, 2015	Mice (C57BL/6J)	Prenatal Alcohol Expos. (PAE)	Ethanol	day 40–55	--	Mitigating “ameliorated”	High Social = Positive
[171]	Clougherty, 2010	Rats (Sprague-Dawley)	Air pollution	Concentrated Ambient fine particles (CAPs) PM_2.5_	--	24	Respiratory response to CAPs exacerbated by chronic stress	Higher Stress = Negative
[7]	Gergs, 2013	Daphnia magna	Alkylphenol	p353-nonylphenol	--	--	Mixed	Higher Stress = Negative
[175]	Gralewicz, 2005	Rats (Wistar)	Organo- phosphate	Chlorfenvinphos	--	--	Protective	Higher Stress = Positive
[4]	McCormick, 2006	Rats (Long-Evans)	Nicotine	Nicotine	30–45 d.	132	Mixed	High Stress (social) = Mixed
[4]	McCormick, 2006	Rats (Long-Evans)	Nicotine	Nicotine	30–45 d.	132	Mixed	Isolation = Mixed
[173]	Reber, 2006	Mice (C57BL/6)	--	--	--	--	--	Higher Stress = Negative

## Supplementary Materials

The Supplementary Materials are available online at https://www.mdpi.com/1660-4601/16/22/4417/s1.

## Acronyms and Abbreviations

ACE	Adverse Childhood Experience(s)
ADHD	Attention Deficit and Hyperactivity Disorder
BMI	Body Mass Index
CAP	Concentrated Ambient Fine Particles
CDC	Centers for Disease Control and Prevention
ETV	Exposure to Violence
Mo.	Month
NO_2_	Nitrogen Dioxide
O_3_	Ozone
PM	Particulate Matter
PRISMA	Preferred Reporting Items for Systematic Reviews and Meta-Analysis
SES/SEP	Socioeconomic Status/Position
SO_2_	Sulfur Dioxide
Yr.	Year
