# Supplementary material for: State-of-the-Science Review of Non-Chemical Stressors Found in a Child’s Social Environment
Source: Int J Environ Res Public Health. 2019 Nov 11;16(22):4417. doi: 10.3390/ijerph16224417 (PMC6888402; doi:10.3390/ijerph16224417)
Supplement: Supplementary file 1 [file ijerph-16-04417-s001.pdf]

Table S1. Summary of the studies included in review.

Acculturation								
	**Author, Year**	**Geographic Location**	**Chemical Stressor**	**Specific Chemical(s)**	**Cohort Age Range**	**Sample Size**	**Impact on Health**
[33]	**Ahn, 2008**	California, USA	--	--	12 - 17 yr.	4,010	High Acculturation = Negative
[34]	**Batis, 2011**	Mexico & USA	--	--	2 - 49 yr.	5,473	High Acculturation = Negative
[51]	**Benbenek, 2012**	Minnesota, USA	--	--	11 - 14 yr.	39	Suggestive
[25]	**Chen, 2011**	San Francisco, CA, USA	--	--	8 - 10 yr.	67	Maternal Low Acculturation = Negative
[24]	**Chen, 2005**	Northern CA, USA	--	--	8 - 10 yr.	68	Maternal Low Acculturation = Negative
[23]	**Chen, 2009**	San Francisco, CA, USA	--	--	8 - 10 yr.	65	Maternal High Acculturation = Positive
[14]	**Dancel, 2015**	USA	--	--	12 mo.	398	Parental Low Acculturation = Negative
[35]	**Dave, 2009**	San Antonio, TX, USA	--	--	5 - 12 yr.	184	High Acculturation = Negative
[26]	**De Maio, 2009**	Canada	--	--	15+ yr.	7,720	Low Acculturation = Negative
[46]	**Elder, 2005**	California, USA	--	--	Mean = 15.5 yr.	106	Mixed
[27]	**Erinosho, 2012**	California, USA	--	--	3 - 5 yr.	1,105	Caregiver Low Acculturation = Negative
[36]	**Hassan, 2012**	Toronto, Canada	--	--	Adults	24	Higher Acculturation = Negative
[37]	**Kaiser, 2015**	Central Valley, CA, USA	--	--	2 - 8 yr.	217	Maternal High Acculturation = Negative
[28]	**Lind, 2012**	Texas, USA	--	--	4th graders (Mean = 9.8 yr.)	3,507	Low Acculturation = Mixed
[38]	**Liu, 2012**	USA	--	--	12 - 19 yr.	2,286	High Acculturation = Negative
[48]	**Martin, 2007**	Chicago, IL, USA	--	--	5 - 12 yr.	336	High Acculturation = Mixed
[39]	**Mazur, 2003**	USA	--	--	4 - 16 yr.	2,985	Low Acculturation = Positive
[50]	**Mendoza, 2014**	Houston, TX, USA	--	--	3 - 5 yr.	96	High Acculturation = None
[47]	**Morello, 2012**	San Diego, CA, USA	--	--	5 - 8 yr.	250	High Acculturation = Mixed
[40]	**Mulasi-Pokhriyal, 2011**	Twin Cities, MN, USA	--	--	9 - 18 yr.	335	Higher Acculturation = Negative
[41]	**Neuhouser, 2004**	Yakima Valley, WA, USA	--	--	18+ yr.	1,689	Higher Acculturation = Negative
[42]	**Nielsen, 2015**	Denmark	--	--	6 mo. - 3.5 yr.	886	Higher Acculturation = Negative

[49]	**Nobari, 2013**	Los Angeles, CA, USA	--	--	2 - 5 yr.	250,029	Acculturation = Mixed
[29]	**Peterman, 2011**	Lowell, MA, USA	--	--	Adults	161	Higher Acculturation = Positive
[43]	**Saint-Jean, 2008**	Florida, USA	--	--	10 - 21 yr.	8,200	Higher Acculturation = Negative
[17]	**Sussner, 2008**	Boston, MA, USA	--	--	23 – 44 yr.	51	Higher Acculturation = Negative
[30]	**Sussner, 2009**	Northeast USA	--	--	24 - 36 mo.	108	Low Acculturation = Negative
[44]	**Unger, 2004**	Southern CA, USA	--	--	6th & 7th grade (Mean ~11 yr.)	2,004	High Acculturation = Negative
[31]	**Velazquez, 2015**	Vancouver, Canada	--	--	grades 5 – 8 (Mean = 12.5 yr.)	950	Low Acculturation = Negative
[45]	**Watt, 2012**	USA	--	--	10 - 17 yr.	37,454	High Acculturation = Negative
[32]	**Wojcicki, 2012**	San Francisco, CA, USA	--	--	grades 4 - 6 (Mean = 10.2 yr.)	144	High Acculturation = Positive
**Adverse Childhood Experiences**							
	**Author, Year**	**Geographic Location**	**Chemical Stressor**	**Specific Chemical(s)**	**Cohort Age Range**	**Sample Size**	**Impact on Health**
[52]	**Anda, 2006**	San Diego, CA, USA	--	--	Adults	17,337	High ACE = Negative
[53]	**Bethell, 2014**	USA	--	--	Birth to 17 yr.	95,677	High ACE = Negative
[54]	**Bright, 2015**	USA	--	--	Birth to 17 yr.	90,555	High ACE = Negative
[198]	**Chilton, 2015**	Philadelphia, PA, USA	--	--	< 4 yr.	31	High ACE = Negative
[56]	**Dong, 2004**	San Diego, CA, USA	--	--	Adults	17,337	High ACE = Negative
[57]	**Dube, 2003**	San Diego, CA, USA	--	--	Adults	17,337	High ACE = Negative
[58]	**Gilbert, 2015**	10 US States and DC	--	--	18+ yr.	53,998	High ACE = Negative
[59]	**Gooding, 2015**	Boston & Cambridge, MA, USA	--	--	13 - 17 yr.	147	High ACE = Negative/None
[60]	**Graham-Bermann, 2005**	Michigan, USA	--	--	4 – 6 yr. (Mean = 4.62)	160	High ACE = Negative
[61]	**Keyes, 2013**	Detroit, MI, USA	--	--	18+ yr.	1,054	High ACE = Negative
[62]	**Lamers-Winkleman, 2012**	Netherlands	--	--	6 - 12 yr.	275	High ACE = Negative
[63]	**Logan-Greene, 2014**	Washington, USA	--	--	18 - 79 yr.	19,333	High ACE = Negative
[64]	**Lyu, 2017**	USA	--	--	25 - 74 yr.	3,366	High ACE = Negative
[65]	**McKelvey, 2017**	Arkansas, USA	--	--	13 - 76 mo.	2,004	High ACE = Negative

[66]	**Peckins, 2012**	USA	--	--	8 - 13 yr.	124	High ACE = Negative
[67]	**Phillips, 2005**	Birmingham, UK	--	--	Mean = 19.8 yr. (±2.3)	57	High ACE = Negative
[68]	**Shalev, 2013**	England	--	--	5 - 10 yr.	236	High ACE = Negative
**Economic**							
	**Author, Year**	**Geographic Location**	**Chemical Stressor**	**Specific Chemical(s)**	**Cohort Age Range**	**Sample Size**	**Impact on Health**
[80]	**Beaver, 2010**	USA	--	--	12 - 21 yr.	~2,000	Low Household income = Negative
[18]	**Berntsson, 2006**	5 Nordic countries (Denmark, Finland, Iceland, Norway, Sweden)	--	--	2 - 17 yr.	20,608	Low Disposable Income = Negative
[92]	**Caughy, 2003**	Baltimore, MD, USA	--	--	3 - 4.5 yr.	200	Neighborhood Income = Inconclusive
[81]	**Chai, 2016**	Global	--	--	Mothers & children	204,159	Higher Income Country = Negative
[82]	**Chen, 2004**	St. Louis, MO, USA	--	--	15 - 19 yr.	100	Low SES = Negative
[83]	**Chen, 2006 (Soc Sci Med)**	USA	--	--	0 - 18 yr.	33,911	Low Income = Negative
[23]	**Chen, 2009**	San Francisco, CA, USA	--	--	8 - 10 yr.	65	High Income = Positive
[84]	**Costa-Font, 2013**	Spain	--	--	2 - 15 yr.	13,358	Income Variation = Negative
[26]	**De Maio, 2009**	Canada	--	--	15+ yr.	7,720	Low SES = Negative
[85]	**Due, 2009**	Denmark	--	--	15 - 27 yr.	614	Low Child SES = Negative
[86]	**Lovasi, 2011**	New York, NY, USA	Pesticides	Chlorpyrifos	36 mo.	266	Neighborhood Poverty = Negative
[39]	**Mazur, 2003**	USA	--	--	4 - 16 yr.	2,985	Household Poverty = Negative
[87]	**Mitchell, 2014**	Urban Cities; USA	--	--	9 yr.	40	Low Income = Negative
[88]	**Nuru-Jeter, 2010**	San Francisco, CA, USA	--	--	8 - 12 yr.	60	Wealth = Positive
[93]	**Prochaska, 2014**	Port Arthur, TX, USA	Exposures from Oil Refineries & Ports	Exposures from Oil Refineries & Ports	Community residents	≈12,028	Low Community SES = Inconclusive
[89]	**Rauh, 2004**	New York, USA	Cigarette smoke	Cigarette smoke	0 - 2 yr.	226	Material Hardship = Negative
[94]	**Schüle, 2016**	Munich, Germany	--	--	5 - 7 yr.	3,499	Low Neighborhood SES = None
[90]	**Seguin, 2007**	Quebec, Canada	--	--	≤ 3.5 yr.	1,950	Poverty Duration = Negative
[31]	**Velazquez, 2015**	Vancouver, Canada	--	--	grades 5 - 8 (Mean = 12.5 yr.)	950	Low Neighborhood SES = Negative

[91]	**Walker, 2015**	USA	--	--	12 - 19 yr.	49	Lower SES = Negative
**Education**							
	**Author, Year**	**Geographic Location**	**Chemical Stressor**	**Specific Chemical(s)**	**Cohort Age Range**	**Sample Size**	**Impact on Health**
[33]	**Ahn, 2008**	California, USA	--	--	12 - 17 yr.	4,010	Low Parental Education = Negative
[81]	**Chai, 2016**	Global	--	--	Mothers & children	204,159	Low Maternal Education = Negative
[102]	**Chen, 2006 (Am J Public Health)**	USA	--	--	0 - 18 yr.	33,911	Low Parental Education = Both
[23]	**Chen, 2009**	San Francisco, CA, USA	--	--	8 - 10 yr.	65	High Maternal Education = Positive
[27]	**Erinosho, 2012**	California, USA	--	--	3 - 5 yr.	1,105	High Caregiver Education = Positive
[96]	**Meara, 2008**	USA	--	--	25 - 84 yr.	--	High Education = Positive
[87]	**Mitchell, 2014**	Urban Cities; USA	--	--	9 yr.	40	Low Maternal Education = Negative
[99]	**Muennig, 2009**	Michigan, USA	--	--	3 - 4 yr.	123	High Maternal Education = Positive
[88]	**Nuru-Jeter, 2010**	San Francisco, CA, USA	--	--	8 - 12 yr.	60	High Family/Caregiver Education = Positive
[29]	**Peterman, 2011**	Lowell, MA, USA	--	--	Adults	161	Higher Education = Positive
[100]	**Protano, 2016**	Latium Region, Italy	--	--	5 - 11 yr.	357	Low Parental Education = Negative
[97]	**Sasson, 2016**	USA	--	--	25+ yr.	US deaths	High Education = Positive
[94]	**Schüle, 2016**	Munich, Germany	--	--	5 - 7 yr.	3,499	Low Parental Education = Negative
[101]	**Walsh, 2007**	Ontario, Canada	--	--	15 - 65 yr.	3,381	Low Education = Negative
**Family**							
	**Author, Year**	**Geographic Location**	**Chemical Stressor**	**Specific Chemical(s)**	**Cohort Age Range**	**Sample Size**	**Impact on Health**
[106]	**Aiyer, 2014**	Flint, MI, USA	--	--	13.9 - 16.9 yr.	266	Presence of Paternal Support = Positive
[80]	**Beaver, 2010**	USA	--	--	12 - 21 yr.	~2,000	Higher Maternal Involvement = Positive (females)
[107]	**Browne, 2012**	Ontario, Canada	--	--	0 - 4 yr.	501	Higher Differential Maternal Negativity = Negative
[24]	**Chen, 2005**	Northern CA, USA	--	--	8 - 10 yr.	68	Poor Family Communication = Negative
[24]	**Chen, 2005**	Northern CA, USA	--	--	8 - 10 yr.	68	Democratic Parenting = Negative

[103]	**Chen, 2017**	Chicago, IL, USA	--	--	9 - 17 yr.	150	Parent-Child Relation = Positive
[108]	**Chen, 2007**	Vancouver, BC, Canada	--	--	9 - 18 yr.	78	Low Family Support = Negative
[84]	**Costa-Font, 2013**	Spain	--	--	2 - 15 yr.	13,358	Mother Employment = Negative (boys)
[109]	**Fabricius, 2007**	Southwestern, USA	--	--	university ages	266	High Father Time = Positive
[71]	**Gonzalez, A, 2012**	Ontario, Canada	--	--	21 - 35 yr.	1,475	Parent Mental Health = Negative
[104]	**Lee, L., 2015**	Boston, MA, USA	--	--	Adults	1,076	Cherished Children = Positive
[110]	**McCracken, 2016**	Edinburgh, UK	--	--	8 - 11 yr.	276	Fewer Siblings = Positive
[105]	**Miller-Graft, 2015**	Sweden	--	--	20 - 24 yr.	703	Parent-Child Warmth = Positive
[87]	**Mitchell, 2014**	Urban Cities; USA	--	--	9 yr.	40	Unstable Family Structure = Negative
[47]	**Morello, 2012**	San Diego, CA, USA	--	--	5 - 8 yr.	250	High Parent Weight = Negative
[16]	**Scharte, 2013**	Bavaria, Germany	--	--	5 - 7 yr.	17,218	Single Parent = Negative
[94]	**Schüle, 2016**	Munich, Germany	--	--	5 - 7 yr.	3,499	High Parent Weight = Negative
[111]	**Turney, 2011**	USA (20 cities)	--	--	Birth - 5 yr.	4,048	Maternal Depression = Negative
[112]	**Waters, 2016**	Monterey, CA, USA	--	--	7 yr.	99	Maternal Depression = Negative
**Food**							
	**Author, Year**	**Geographic Location**	**Chemical Stressor**	**Specific Chemical(s)**	**Cohort Age Range**	**Sample Size**	**Impact on Health**
[113]	**Baer, 2015**	Boston, MA, USA	--	--	15 - 25 yr.	400	Higher Insecurity = Negative
[25]	**Chen, 2011**	San Francisco, CA, USA	--	--	8 - 10 yr.	67	Poor Food Choices = Negative
[35]	**Dave, 2009**	San Antonio, TX, USA	--	--	5 - 12 yr.	184	High Food Insecure = Negative
[143]	**Drewnowski, 2012**	King County, WA, USA	--	--	Adults	1,682	Lower-Price Market = Negative
[120]	**Grin, 2013**	Miami, FL, USA	--	--	18+ yr. mothers	181	Lower Food Security = Negative
[37]	**Kaiser, 2015**	Central Valley, CA, USA	--	--	2 - 8 yr.	217	Meal Skip/Pattern = Negative
[138]	**Kaur, 2015**	USA	--	--	2 - 11 yr.	9,701	Food Insecurity Age 6-11 = Negative
[138]	**Kaur, 2015**	USA	--	--	2 - 11 yr.	9,701	Food Insecurity Age 2-5 = None
[38]	**Liu, 2012**	USA	--	--	12 - 19 yr.	2,286	Poor Diet = Negative
[142]	**Lumia, 2015**	Finland	Cow's milk[1]	--	0 – 6 yr.	900	Early Fish Consumption = Positive
[139]	**Melchior, 2012**	Quebec, Canada	--	--	1½ - 8 yr.	2,120	Food Insecurity = Negative

[93]	**Prochaska, 2014**	Port Arthur, TX, USA	Exposures from Oil Refineries & Ports	Exposures from Oil Refineries & Ports	Community residents	≈12,028	Food Access = Inconclusive
[140]	**Ryu, 2012**	USA	--	--	Kindergarten to 8th grade	7,326	Persistent Food Insecurity = Negative
[141]	**Spiers, 2016**	Illinois, USA	--	--	2 - 5 yr.	438	Food Insecure = Inconclusive
[132]	**Taylor, 2012**	Prince Edward Island, Canada	--	--	5th & 6th grade	1,980	School (vs home) lunch = Inconclusive
[31]	**Velazquez, 2015**	Vancouver, Canada	--	--	grades 5 – 8 (Mean = 12.5 yr.)	950	Food Insecurity = Negative
[32]	**Wojcicki, 2012**	San Francisco, CA, USA	--	--	grades 4 - 6 (Mean = 10.2 yr.)	144	Eat Breakfast = Positive
**Greenspace/(Bluespace)**							
	**Author, Year**	**Geographic Location**	**Chemical Stressor**	**Specific Chemical(s)**	**Cohort Age Range**	**Sample Size**	**Impact on Health**
[103]	**Chen, 2017**	Chicago, IL, USA	--	--	9 - 17 yr.	150	Residential Greenspace = Positive
[144]	**Hordyk, 2015**	Montreal, Quebec, Canada	--	--	7 - 13 yr.	7 Adults 13 Child.	Contact with Urban Greenspace = Positive
[110]	**McCracken, 2016**	Edinburgh, UK	--	--	8 - 11 yr.	276	Greenspace Use = Positive
[110]	**McCracken, 2016**	Edinburgh, UK	--	--	8 - 11 yr.	276	Residential Greenspace = None
[145]	**Ou, 2016**	Chelsea, MA, USA	--	--	18+ yr.	354	Proximity to Preferred Park = Positive
[94]	**Schüle, 2016**	Munich, Germany	--	--	5 - 7 yr.	3,499	Perceived Lack of Greenspace = Negative
[146]	**Wood, 2016**	England	--	--	10 - 11 yr.	1,475,617	Proximity to Bluespace (coast) = Inconclusive
[147]	**Younan, 2016**	Southern CA, USA	--	--	9 - 18 yr.	1,287	Greenspace (Neighborhood) = Positive
**Neighborhood**							
	**Author, Year**	**Geographic Location**	**Chemical Stressor**	**Specific Chemical(s)**	**Cohort Age Range**	**Sample Size**	**Impact on Health**
[80]	**Beaver, 2010**	USA	--	--	12 - 21 yr.	≈2,000	Neighborhood Disadvantage = None
[108]	**Chen, 2007**	Vancouver, BC, Canada	--	--	9 - 18 yr.	78	Greater Neighborhood Problems = Negative
[150]	**Curtis, 2004**	Canada	--	--	4 - 11 yr.	11,037	Low Neighborhood Quality = Negative
[149]	**Fagan, 2015**	Chicago, IL, USA	--	--	9 – 19 yr. (Mean = 13.94)	1,416	Higher Neighborhood Disadvantage = Negative
[50]	**Mendoza, 2014**	Houston, TX, USA	--	--	3 – 5 yr.	96	Higher Neighborhood Disorder = Negative

[93]	**Prochaska, 2014**	Port Arthur, TX, USA	Exposures from Oil Refineries & Ports	Exposures from Oil Refineries & Ports	Community residents	≈12,028	EJ Community = Inconclusive
[148]	**Shmool, 2014**	New York, NY, USA	Air pollution	Black carbon, NO_2, O_3, PM2.5, SO_2	0 - 14 yr.	NYC residents	High Physical Disorder (& violent crime) = Negative
[147]	**Younan, 2016**	Southern CA, USA	--	--	9 - 18 yr.	1,287	Neighborhood Quality = None
				**Social**			
	**Author, Year**	**Geographic Location**	**Chemical Stressor**	**Specific Chemical(s)**	**Cohort Age Range**	**Sample Size**	**Impact on Health**
[18]	**Berntsson, 2006**	5 Nordic countries (Denmark, Finland, Iceland, Norway, & Sweden)	--	--	2 - 17 yr.	20,608	High Social Capital = Positive
[156]	**Borgonovi, 2010**	Britain	--	--	Adults	35,000	High Social Capital = Positive
[107]	**Browne, 2012**	Canada	--	--	0 - 4 yr.	501	High Social Disadvantage (Familial) = Negative
[157]	**Caldwell, 2015**	New Mexico, USA	Prenatal alcohol exposure	Ethanol	day 40–55		High Social = Positive
[92]	**Caughy, 2003**	Baltimore, MD, USA	--	--	3 - 4.5 yr.	200	Low Parental Social = Inconclusive
[164]	**Chandola, 2007**	Britain	--	--	50 - 74 yr.	6,944	Lack/Negative Social Support = Negative
[82]	**Chen, 2004**	St. Louis, MO, USA	--	--	15 - 19 yr.	100	Large Social Environment = Positive
[108]	**Chen, 2007**	Vancouver, BC, Canada	--	--	9 - 18 yr.	78	Peer-Support = None
[152]	**Cheng, 2014**	Baltimore, MD, USA; New Delhi, India; Ibadan, Nigeria; Johannesburg, South Africa; and Shanghai, China	--	--	15 - 19 yr.	2,393	High Social = Positive
[167]	**Evans-Polce, 2016**	Britain	--	--	Adults	11,469	Social Support = None
[104]	**Lee, L., 2015**	Boston, MA, USA	--	--	Adults	1,076	High Social Support = Positive
[154]	**Lee, C., 2016**	Northeastern, USA	--	--	18 - 25 yr.	636	High Social Support = Positive
[63]	**Logan-Greene, 2014**	Washington, USA	--	--	18 - 79 yr.	19,333	High Social = Positive
[151]	**Lynch-Jordan, 2015**	USA	--	--	Mean = 21.6 yr. (±1.9)	127	Higher Perceived Social Support = Positive

[64]	**Lyu, 2017**	USA	--	--	24 - 74 yr.	3,366	Low Quality Social Relations = Negative
[158]	**McConnell, 2010**	Alberta, Canada	--	--	0 - 17 yr.	923	Higher Parental Social Support = Positive
[110]	**McCracken, 2016**	Edinburgh, UK	--	--	8 - 11 yr.	276	Higher Friends = Positive
[159]	**Mohnen, 2012**	Netherlands	--	--	18+ yr.	65,990	Neighborhood Social Capital = Positive
[165]	**Non, 2016**	Boston, MA; Providence, RI, USA	--	--	Adults (age 7 when soc. disadvantage)	564	High Childhood Social Disadvantage = Negative
[67]	**Phillips, 2005**	Birmingham, UK	--	--	Mean = 19.8 yr. (±2.3)	57	Higher Social Support = Positive
[166]	**Razani, 2015**	USA	--	--	6 - 17 yr.	64,076	Lower Neighborhood Support = Negative
[90]	**Seguin, 2007**	Quebec, Canada	--	--	≤ 3.5 yr.	1,950	Social Support, Mother = None
[17]	**Sussner, 2008**	Boston, MA, USA	--	--	23 - 44 yr.	51	Low Social = Negative
[160]	**Tomfohr, 2015**	San Diego, CA, USA	Poor sleep[1]	--	Adult (Mean = 46.3 yr.)	67	High Social = Positive
[91]	**Walker, 2015**	USA	--	--	12 - 19 yr.	49	High Social Support = Positive
[45]	**Watt, 2012**	USA	--	--	10 - 17 yr.	37,454	High Parental Social = Positive
[162]	**Widom, 2015**	Midwestern USA	--	--	0 - 11 yr.	908	Social Support = None
[155]	**Woodgate, 2006**	Western Canada	--	--	12 - 18 yr.	15	High Social Support = Positive
[163]	**Ziv, 2012**	USA, (metropolitan)	--	--	48 - 63 mos.	256	High Perception Social = Positive
**Stress**							
	**Author, Year**	**Geographic Location**	**Chemical Stressor**	**Specific Chemical(s)**	**Cohort Age Range**	**Sample Size**	**Impact on Health**
[168]	**Bair-Merritt, 2012**	USA	--	--	< 18 yr.	55	Higher Stress = Negative
[169]	**Chen, 2008**	Vancouver, BC, Canada	Traffic pollution	NO_2	9 - 18 yr.	73	Higher Stress = Negative
[170]	**Clougherty, 2007**	Boston, MA, USA	Air pollution	NO_2	Birth – 12 yr. (Mean = 6.8 yr.)	413	Higher Stress = Negative
[171]	**Clougherty, 2010**	--	Air pollution	Concentrated ambient fine particles (CAPs) PM2.5		24	Higher Stress = Negative
[172]	**Cowell, 2015**	Boston, MA, USA	Prenatal exposure to traffic-related air pollution	Black carbon	6 yr.	258	Higher Stress = Negative

[7]	**Gergs, 2013**	_--	Alkylphenol	p353-nonylphenol	--	--	Higher Stress = Negative
[60]	**Graham-Bermann, 2005**	Michigan, USA	--	--	4 – 6 yr. (Mean = 4.62)	160	Higher Stress = Negative
[175]	**Gralewicz, 2005**	(Rats) Poland	Organophosphate pesticide	Chlorfenvinphos	--	~30	Higher Stress = Positive
[48]	**Martin, 2007**	Chicago, IL, USA	--	--	5 - 12 yr.	336	Higher Caregiver Stress = Negative
[4]	**McCormick, 2007**	(Rats) Canada	Nicotine	Nicotine	30 - 45 d.	132	High Stress (social) = Mixed
[67]	**Phillips, 2005**	Birmingham, UK	--	--	Mean 19.8 yr. (±2.3)	57	Stressful Life Events = Negative
[173]	**Reber, 2006**	--	--	--	--	97	Higher Stress = Negative
[68]	**Shalev, 2013**	England	--	--	5 - 10 yr.	236	Higher Stress = Negative
[174]	**Wright, 2004**	Boston, Chicago, New York City, Dallas, Seattle, & Tucson.	--	--	5 - 12 yr.	851	Caregiver-perceived Stress = Inconclusive
**Urbanicity**							
	**Author, Year**	**Geographic Location**	**Chemical Stressor**	**Specific Chemical(s)**	**Cohort Age Range**	**Sample Size**	**Impact on Health**
[180]	**Breslau, 2004**	Mid-Atlantic city, USA	--	--	19 - 24 yr.	1,698	High Urban= Suggested/Inconclusive
[81]	**Chai, 2016**	Global	--	--	Mothers & children	204,159	High Urban = Negative
[27]	**Erinosho, 2012**	California, USA	--	--	3 - 5 yr.	1,105	High Rural = Negative
[100]	**Protano, 2016**	Latium Region, Italy	--	--	5 - 11 yr.	357	High Rural = Negative
[146]	**Wood, 2016**	England	--	--	10 - 11 yr.	1,475,617	High Urban = Negative
**Exposure to Violence (ETV)**							
	**Author, Year**	**Geographic Location**	**Chemical Stressor**	**Specific Chemical(s)**	**Cohort Age Range**	**Sample Size**	**Impact on Health**
[106]	**Aiyer, 2014**	Flint, MI, USA	--	--	13.9 - 16.9 yr.	266	Higher ETV = Negative
[195]	**Bair-Merritt, 2015**	NC & PA, USA	--	--	7 - 48 mo.	961	Higher Maternal ETV = Negative
[168]	**Bair-Merritt, 2012**	USA	--	--	< 18 yr.	55	Higher ETV = Negative
[70]	**Boynton-Jarrett, 2008**	USA	--	--	12 - 18 yr.	8,224	Higher ETV = Negative
[196]	**Boynton-Jarrett, 2013**	USA	--	--	12 - 14 yr.	4,834	Higher ETV = Negative
[180]	**Breslau, 2004**	Mid-Atlantic city, USA	--	--	19 - 24 yr.	1,698	Higher ETV = Negative

[197]	**Buckner, 2004**	Massachusetts, US	--	--	8 - 17 yr.	95	Higher ETV = Negative
[81]	**Chai, 2016**	Global	--	--	Mothers & children	204,159	Higher Maternal ETV = Negative
[198]	**Chilton, 2013**	Philadelphia, PA, USA	--	--	< 4 yr.	44	Higher Maternal ETV = Negative
[181]	**Chiu, 2014**	Boston, MA, USA	Urban Pollution	Black carbon, PM2.5	2 yr.	708	Community Violence = Negative
[170]	**Clougherty, 2007**	Boston, MA, USA	Air pollution	NO_2	Birth – 12 yr. (Mean = 6.8 yr.)	413	Above Median ETV = Negative
[56]	**Dong, 2004**	San Diego, CA, USA	--	--	Adults	17,337	Higher ETV = Negative
[199]	**Dowdell, 2012**	Philadelphia, PA, USA	--	--	11 - 13 yr.	379	Higher ETV = Negative
[85]	**Due, 2009**	Denmark	--	--	15 - 27 yr.	614	High ETV (Bullying) = Negative
[149]	**Fagan, 2015**	Chicago, IL, USA	--	--	9 – 19 yr. (Mean = 13.94)	1,416	Higher ETV = Negative
[200]	**Franzese, 2014**	USA	--	--	11 - 17 yr.	2,360	Higher ETV = Negative
[71]	**Gonzalez, A, 2012**	Ontario, Canada	--	--	21 - 35 yr.	1,475	Higher ETV = Negative
[59]	**Gooding, 2015**	Boston & Cambridge, MA, USA	--	--	13 - 17 yr.	147	ETV = Negative & None
[60]	**Graham-Bermann, 2005**	Michigan, USA	--	--	4 – 6 yr. (Mean = 4.62)	160	Higher ETV = Negative
[72]	**Halpern, 2013**	USA	--	--	11 - 21 yr.	8,531	Higher ETV = Negative
[201]	**Haynie, 2008**	USA	--	--	grades 7 - 12	11,949	Higher ETV = Negative
[202]	**Kacanek, 2016**	USA & Puerto Rico	--	--	8 - 15 yr.	268	Higher ETV = Negative
[203]	**Kliewer, 2015**	Philadelphia, PA & Richmond, VA, USA	--	--	11 - 14 yr.	362	Higher ETV = Negative
[62]	**Lamers-Winkleman, 2012**	Netherlands	--	--	6 - 12 yr.	275	Higher ETV = Negative
[204]	**Matthews, 2017**	Pittsburgh, PA, USA	--	--	30 - 34 yr.	305	Higher Bullying = Negative
[105]	**Miller-Graft, 2015**	Sweden	--	--	20 - 24 yr.	703	Higher ETV = Negative
[145]	**Ou, 2016**	Chelsea, MA, USA	--	--	18+ yr.	354	High Known ETV = Negative
[189]	**Oulette-Morin, 2016**	Quebec, Canada	--	--	21 yr.	327	Higher ETV = Negative
[66]	**Peckins, 2012**	USA	--	--	8 - 13 yr.	124	Higher ETV = Negative
[191]	**Ragavan, 2016**	Northern California, USA	--	--	Youths (13-17 yr.), Women (≥18 yr.)	17<br>46	Inconclusive
[73]	**Riley, E., 2010**	From 14 US States	--	--	Adults	68,505	Higher ETV = Negative

[192]	**Rosas-Salazar, 2016**	Puerto Rico & CT, USA	--	--	9 - 14 yr.	747	Higher ETV = Negative
[68]	**Shalev, 2013**	England	--	--	5 - 10 yr.	236	Higher ETV = Negative
[148]	**Shmool, 2014**	New York, NY, USA	Air pollution	Black carbon, NO_2, O_3, SO_2, PM2.5	0 - 14 yr.	NYC residents	Higher Violent Crime (and physical disorder)[2] = Negative
[193]	**Sourander, 2007**	Finland	--	--	18 - 23 yr.	2,540	Higher Bullying = Negative
[190]	**Sternthal, 2010**	Chicago, IL, USA	--	--	0 - 9 yr.	2,071	Higher ETV = Negative
[194]	**Suglia, 2009**	Boston, MA, USA	--	--	7 - 13 yr.	43	Higher ETV = Negative
[161]	**Walker, 2008**	--	--	--	5 - 12 yr.	231	Higher ETV = Negative
[101]	**Walsh, 2007**	Ontario, Canada	--	--	15 - 65 yr.	3,381	Physical ETV = Negative
[101]	**Walsh, 2007**	Ontario, Canada	--	--	15 - 65 yr.	3,381	Sexual ETV = None
[162]	**Widom, 2015**	Midwestern USA	--	--	0 - 11 yr.	908	Higher ETV = Negative
[174]	**Wright, 2004**	Boston, Chicago, New York City, Dallas, Seattle, & Tucson.	--	--	5 - 12 yr.	851	Higher ETV = Negative
[163]	**Ziv, 2012**	USA, (metropolitan)	--	--	48 - 63 mos.	256	Higher ETV = Negative
**Other**							
	**Author, Year**	**Geographic Location**	**Chemical Stressor**	**Specific Chemical(s)**	**Cohort Age Range**	**Sample Size**	**Impact on Health**
[33]	**Ahn, 2008**	California, USA	--	--	12 - 17 yr.	4,010	Race = Negative
[80]	**Beaver, 2010**	USA	--	--	12 - 21 yr.	~2,000	Longer Breastfeeding = Positive
[80]	**Beaver, 2010**	USA	--	--	12 - 21 yr.	~2,000	Household Smoking = Negative
[196]	**Boynton-Jarrett, 2013**	USA	--	--	12 - 14 yr.	4,834	High Turbulence = Negative
[180]	**Breslau, 2004**	Mid-Atlantic city, USA	--	--	19 - 24 yr.	1,698	City "area" = Negative
[102]	**Chen, 2006 (Am J Public Health)**	USA	--	--	0 - 18 yr.	33,911	Ethnicity = Both
[26]	**De Maio, 2009**	Canada	--	--	15+ yr.	7,720	High Discrimination = Negative
[143]	**Drewnowski, 2012**	King County, WA, USA	--	--	Adults	1,682	Distance to Market = None
[98]	**Levin, 2013**	Midwest, USA	--	--	1 - 13 yr.	104	High Household Chaos = Negative
[38]	**Liu, 2012**	USA	--	--	12 - 19 yr.	2,286	Low Activity = Negative
[64]	**Lyu, 2017**	USA	--	--	24 - 74 yr.	3,366	High Child Misfortune = Negative

[4]	**McCormick, 2007**	(Rats) Canada	Nicotine	Nicotine	30 - 45 d.	132	Isolation = Mixed
[50]	**Mendoza, 2014**	Houston, TX, USA	--	--	3 - 5 yr.	96	Lower Physical Activity = Negative
[192]	**Rosas-Salazar, 2016**	Puerto Rico & CT, USA	--	--	9 - 14 yr.	747	African Ancestry = Negative
[148]	**Shmool, 2014**	New York, NY, USA	Air pollution	Black carbon, NO_2, O_3, PM2.5, SO_2	0 - 14 yr.	NYC residents	Crowding/No Resource = Negative
[160]	**Tomfohr, 2015**	San Diego, CA, USA	Poor sleep[1]	--	Adults (Mean = 46.3 yr.)	67	Poor Sleep = Negative
[112]	**Waters, 2016**	Monterey, CA, USA	--	--	7 yr.	99	Overcrowding = None
[45]	**Watt, 2012**	USA	--	--	10 - 17 yr.	37,454	Ethnicity = Negative
[32]	**Wojcicki, 2012**	San Francisco, CA, USA	--	--	grades 4 - 6 (Mean = 10.2 yr.)	144	Latin Country of Origin. = Negative

[1] Cow's milk and poor sleep are not true chemical stressors; but, were considered as a factor in the design that was influenced by the non-chemical stressor.

[2] Shmool et al [148] measured for violent crime and neighborhood disorder as a combined indicator. These findings are calculated in both the neighborhood category and the exposure to violence category, which may result in a slight overestimation in each category.
